# Worldwide research trends on aristolochic acids (1957–2017): Suggestions for researchers

**DOI:** 10.1371/journal.pone.0216135

**Published:** 2019-05-02

**Authors:** Qiang Zhou, Jin Pei, Josiah Poon, Alexander Y. Lau, Li Zhang, Yuhua Wang, Chang Liu, Linfang Huang

**Affiliations:** 1 Engineering Research Center of Chinese Medicine Resource, Ministry of Education, Institute of Medicinal Plant Development, Chinese Academy of Medical Sciences & Peking Union Medical College, Beijing, China; 2 College of Pharmacy, Chengdu University of Traditional Chinese Medicine, Chengdu, Sichuan, China; 3 School of Information Technologies, The University of Sydney, Sydney, Australia; 4 Analytic and Clinical Cooperative Laboratory of Integrative Medicine, Chinese University of Hong Kong and The University of Sydney, Sydney, Australia; 5 Department of Medicine and Therapeutics, Chinese University of Hong Kong, Hong Kong SAR, China; 6 College of Science, Sichuan Agricultural University, Yaan, Sichuan, China; 7 College of Pharmacy, Inner Mongolia Medical University, Hohhot, Inner Mongolia, China; University of Toronto, CANADA

## Abstract

Aristolochic acids and their derivatives are components of many traditional medicines that have been used for thousands of years, particularly in Asian countries. To study the trends of research into aristolochic acids and provide suggestions for future study, we performed the following work. In this paper, we performed a bibliometric analysis using CiteSpace and HistCite software. We reviewed the three phases of the development of aristolochic acids by using bibliometrics. In addition, we performed a longitudinal review of published review articles over 60 years: 1,217 articles and 189 review articles on the history of aristolochic acid research published between 1957 and 2017 were analyzed. The performances of relevant countries, institutions, and authors are presented; the evolutionary trends of different categories are revealed; the history of research into aristolochic acids is divided into three phases, each of which has unique characteristics; and a roadmap of the historical overview of aristolochic acid research is finally established. Finally, five pertinent suggestions for future research into aristolochic acid are offered: (1) The study of the antitumor efficacy of aristolochic acids is of value; (2) The immune activity of aristolochic acids should be explored further; (3) Researchers should perform a thorough overview of the discovery of naturally occurring aristolochic acids; (4) More efforts should be directed toward exploring the correlation between aristolochic acid mutational signature and various cancers; (5) Further efforts should be devoted to the research and review work related to analytical chemistry. Our study is expected to benefit researchers in shaping future research directions.

## Introduction

Aristolochic acids (AAs) and their derivatives have attracted worldwide attention owing to concerns about their safety. In October 2017, an article published in *Science Translational Medicine* indicated that AAs and their derivatives were implicated widely in liver cancer in Taiwan and throughout Asia [1], which once again raised questions on the safety of traditional medicines containing AAs and their derivatives. During their application, reports of adverse effects on kidney failure and urothelial cancers related to AAs have continued to emerge [2–7]. In the early 1990s, inadvertent treatment with AA-containing herbs at a weight-loss clinic in Belgium resulted in kidney failure in approximately 100 women [8, 9]. In the early 20^th^ century in China, an incident with Gentian purging liver pills (which once contained *Aristolochia manshuriensis*) triggered widespread panic about the safety of traditional Chinese medicines [10]. In 2007, scientists claimed that advances in the understanding of endemic nephropathy favored the causative role of AA in endemic (Balkan) nephropathy [11]. Thus, the use of AA-containing herbal remedies has been gradually prohibited in many countries and regions [12–14]. In 2001, the Food and Drug Administration issued warnings and an import alert that herbal products were unsafe if they contained or were suspected to contain AA [15]. In 2012, the International Agency for Research on Cancer, the WHO cancer agency, categorized AA I, AA II, and aristolactam as proven group 1 human carcinogens [16]. It should be noted that, although AA-containing plants have been used in China for thousands of years in traditional prescriptions, the Chinese government banned the use of AA-containing herbs for safety reasons and removed them from the Chinese Pharmacopoeia in 2003. At the end of October 2017, the China Food and Drug Administration produced lists in which they indicated 43 Chinese patent medicines and 24 Aristolochiaceae herbs that may contain AA (http://samr.cfda.gov.cn/WS01/CL1991/215893.html; http://samr.cfda.gov.cn/WS01/CL1991/215894.html). Owing to the contradiction between the long history of traditional applications and the well-defined toxicity, a panorama of AA research is needed to identify the trends in the research into AA in various domains and to determine any scientific issues that have not been fully explored.

We conducted a bibliometric analysis and performed a comprehensive review of AA research. Bibliometrics, the application of statistics and mathematics for the analysis of written publications, such as books and journal articles, is now used routinely to evaluate large numbers of scientific articles in a given research domain [17–21]. To identify important drivers and research trends at the interface between medicine and food science, Yeung *et al*. conducted a bibliometric analysis of the 100 most-cited articles in ethnopharmacology and indicated that this analysis highlighted the emerging importance in the context of disease prevention (food science), but also in the development of research driven by the needs and interests of the fast developing economies, most notably in Asia [22]. To measure and understand the scope of practice on the history of modern drug discovery, Baker *et al*. conducted a bibliometric analysis of drug repurposing [23]. Aggarwal *et al*. used bibliometrics to analyze global lung cancer research between 2004 and 2013, and established that, relative to the huge health, social, and economic burden associated with lung cancer, the level of world research output lags significantly behind that of research into other malignancies [24].

The aim of this study was to use bibliometric techniques to provide a 60-year longitudinal view (1957 to 2017) of the evolution of the scientific literature on AA without focusing on a specific area. Two software programs were employed: CiteSpace and HistCite. Publication activities were investigated based on the following aspects: year of publication, country, institution, author, research domain, keyword, article, and journal. Finally, the results of bibliometric analysis and a traditional review conducted under the guidance of bibliometrics were integrated to divide the history of AA research into three phases and clearly present the evolution of AA research. A roadmap that presents a panorama of AA research history was established. Relevant review articles were also analyzed. Based on the above work, five clear suggestions are provided. This work is the first attempt to compile scientific research on AA. It is expected to benefit researchers by shaping future research directions and providing references for policy formulation.

## Materials and methods

**Fig 1:**
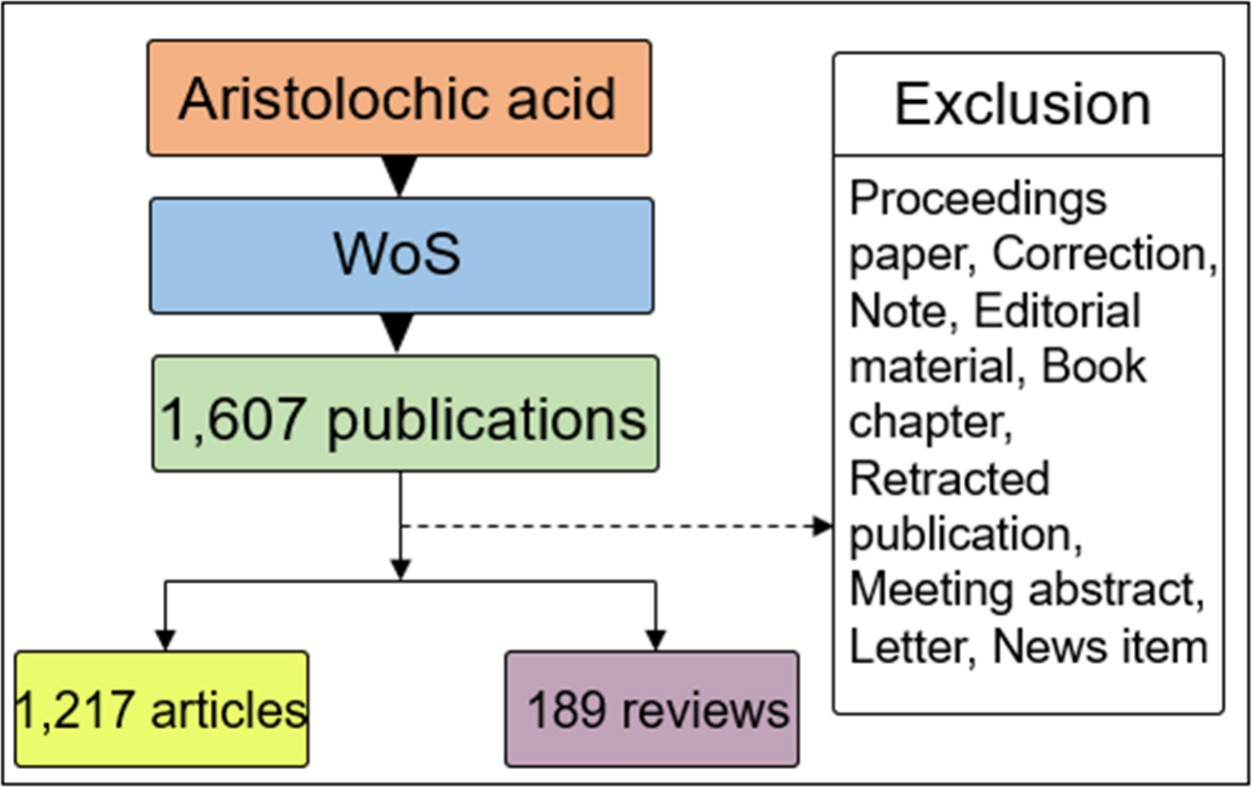
Retrieval strategy and number of publications.

The Thomson-Reuter’s Web of Science (WoS) was used to compile the literature dataset because this database provides a comprehensive and standardized set of data for export and has been used extensively in academia. The first article on AA was published in 1957; thus, the timespan for the retrieval of the experiments in this study was from 1957 to 2017 to investigate the global scientific trends in AA research over a long period. The search was conducted in January 2018. In the initial step of the bibliometric literature analysis, we used “aristolochic acid” as the search term in the WoS database and retrieved 1,603 documents. As part of the search, we performed several queries in succession, such as using “aristolochic acids” or “aristolochic acids and derivatives” as the search term. Finally, we used “aristolochic acid” as the retrieval term because it led to almost every relevant search result. From the different types of relevant literature (e.g., articles, proceedings, reviews, and book chapters), only research articles and review papers were selected for further analysis. Finally, 1,217 articles and 189 reviews were analyzed. Almost all the obtained articles were in English (1,174 or 96.5%); the others were in nine different languages, mainly German (14 or 1.1%), Chinese (11 or 0.9%), and French (11 or 0.9%). The retrieval strategy of the experiments is shown in Fig 1.

The data were independently extracted from all qualifying publications by authors. The text data downloaded from WoS were imported into CiteSpace and were quantitatively and qualitatively analyzed. Bibliometric indicators were extracted from the data, including publication number, (total) local citation score (TLCS and LCS), (total) global citation score (TGCS and GCS), and h-index. The h-index is another alternative indicator used to evaluate the quality of research publications depending on the number of citations received.

In this study, network analysis was conducted to clarify the relationship among different items by an underlying network of node links through which information or social relationships travel. Such a network can help to evaluate the importance and influence of a node through the measurement of the centrality of the nodes. Among the various software tools, CiteSpace is frequently used for the visualization of networks and to perform bibliographic coupling analysis. HistCite is another software tool used to perform bibliometric analysis and identify relevant indicators. In this study, bibliometric literature analysis was performed using various types of indicators, such as descriptive (year of publication, subject categories, authors), relational (collaborations among countries, institutions), and qualitative (citations, impact factors, h-index). After data mining procedures using a bibliometric approach, a traditional literature review of the important studies and reviews on the history of AA research was performed in this study.

## Results and discussion

### Characteristics of countries

**Fig 2:**
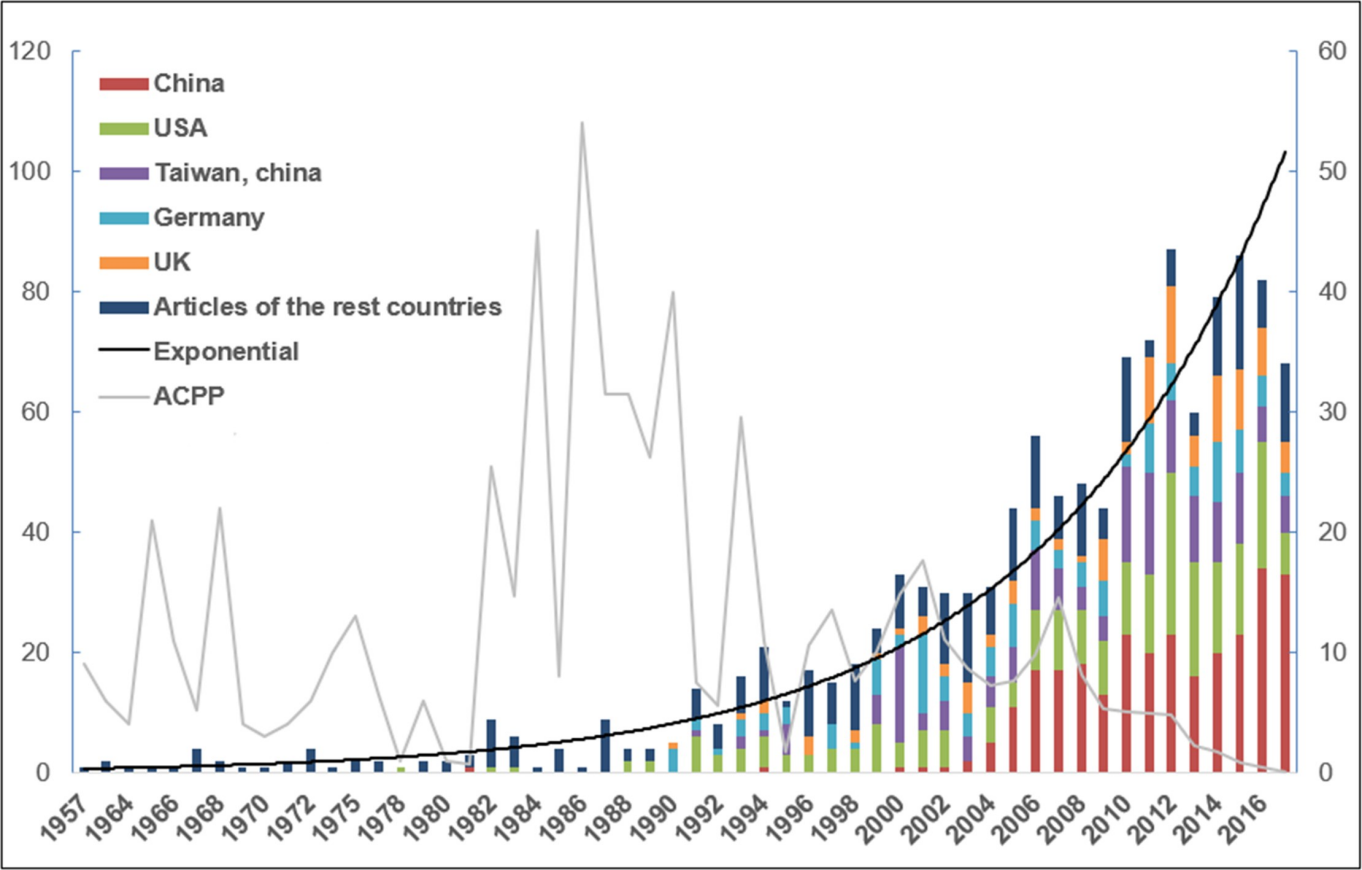
Worldwide output from 1957 to 2017. The left axis represents the total number of articles per year and the right axis represents the average citation per article per year (ACPP).

**Fig 3:**
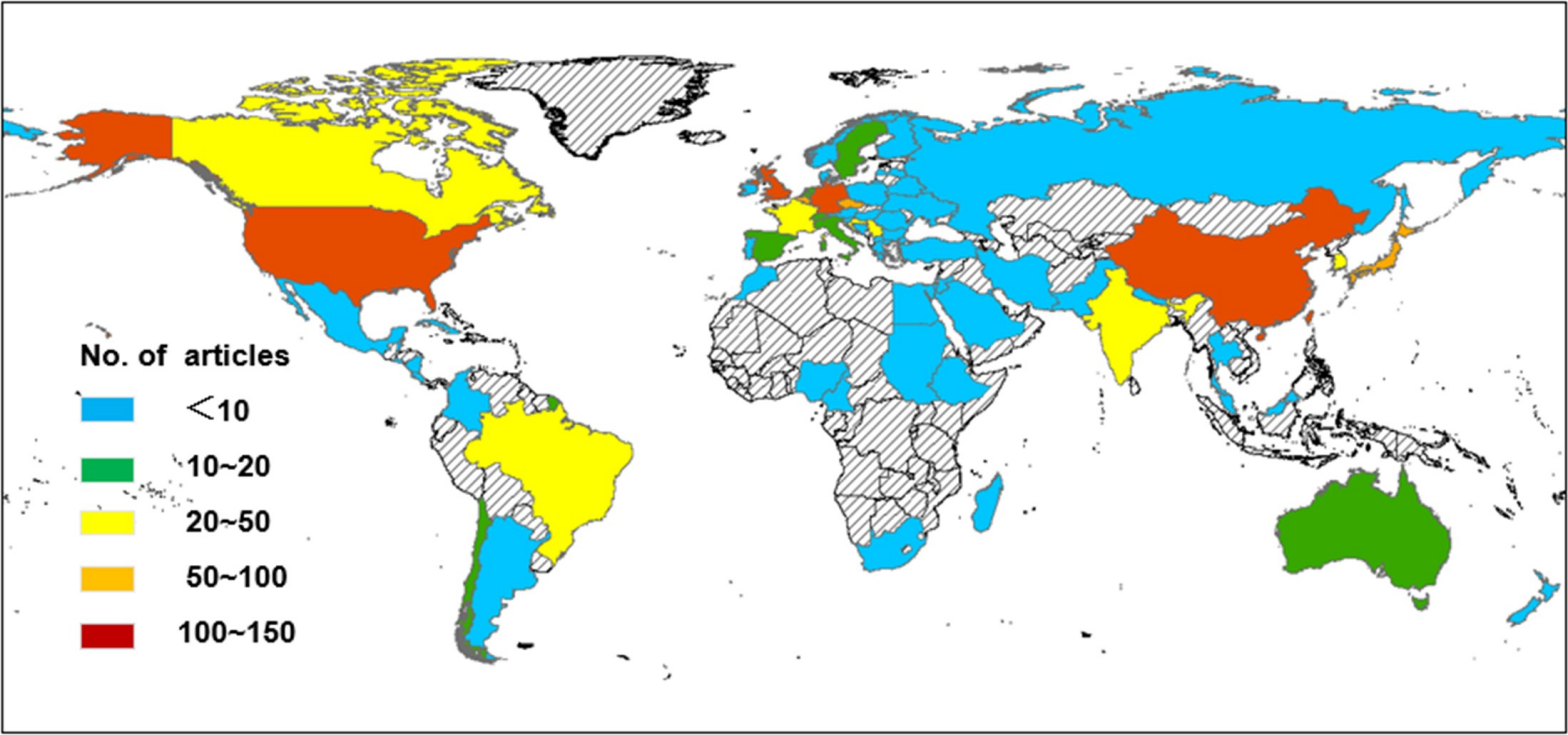
Geographical distribution of AA-related research articles, 1957–2017.

Three quarters of the 1,217 articles came from the top five countries (Fig 2). China (excluding the Taiwan Region and Hong Kong) published the most papers (280, 23.0%), followed by the United States (246, 20.2%), the Taiwan Region (168, 13.8%), Germany (127, 10.4%), and the UK (105, 8.6%). The geographical distribution of the total articles on AA research from all the countries and regions is illustrated in Fig 3. Publications are distributed throughout the world, although several areas have lower output.

**Fig 4:**
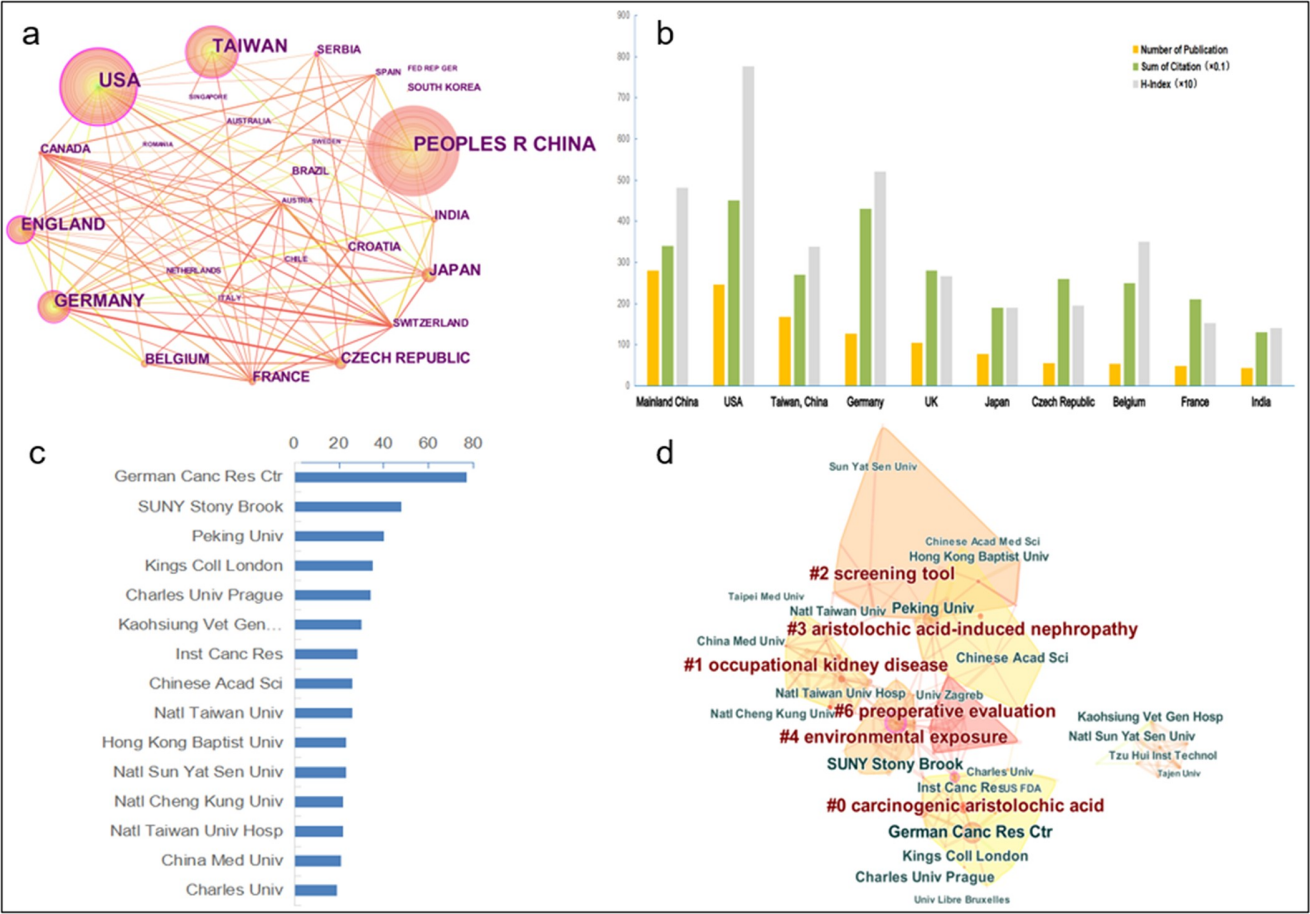
Characteristics of cooperation between countries and performances of institutions. A network representation of the intensity of the collaboration between countries and regions (a); The distribution, citation frequency (×0.1) and h-index (×10) of publications in the top 10 countries and regions (b); The number of articles on AA research from the top 15 most productive institutions (1957–2017) (c); The international collaborations among the most productive institutions and the bibliographic coupling analysis (d). Each country is presented as a node: the depth of the node color is proportional to the total number of collaborations; the size of the node reflects the outputs of countries or regions; and the thickness related to the node demonstrates the strength of collaborations.

The international collaborations among the 20 most productive countries and regions were visualized by using CiteSpace, as shown in Fig 4A. Cooperation between the countries of Switzerland–Austria ranked first, followed by Germany–Czech Republic, England–Belgium, Switzerland–Italy, and Germany–India. In the most productive countries and regions, the United States, Germany, England, Japan, and Czech Republic maintained active collaborations with the rest of the countries, whereas China, Taiwan Region and Belgium had a large number of publications but lacked international collaborations. Subsequently, we compared the performance of document co-citation and h-index of the top 10 most productive countries. As shown in Fig 4B, China ranked first in the article production, but had a relatively low number of total citations and h-index. Despite Germany and Belgium producing fewer publications, their total citations and h-index values were higher than many other productive countries, which was indication of the high quality of these publications. The United States was ahead in overall strength, second in outputs, and highest in total citations and h-index values.

### Performance of institutions

The rankings of the top 15 most productive institutions are presented in Fig 4C. The highest ranked, by a large margin, was the German Cancer Research Center (German Canc Res Ctr; 77, 6.3%), followed by the State University of New Your (SUNY) at Stony Brook, United States (48, 3.9%), and Peking University (40, 3.3%). The total publications from Chinese institutions ranked first (133, 109%), which was unsurprising as five institutions were located in China (including universities and research institutes). This result indicated that the Chinese research institutions were more active in such a field than institutions in other countries. The Taiwan region ranked second in terms of outputs (100, 8.2%).

The international collaborations among the most productive institutions and the bibliographic coupling analysis are shown in Fig 4D. The clustering result contains seven clusters, and the silhouette among the different clusters was clear, except for Cluster #5 on the right. These clusters were labeled by index terms from their own publications. The largest two clusters were summarized by using CiteSpace. The largest cluster (#0) has 16 members, a silhouette value of 0.789 and is labeled as *carcinogenic aristolochic acid* by LLR (log-likelihood ratio). The most cited author in the cluster is Arlt (2011), who wrote *Role of p450 1a1 and p450 1a2 in Bioactivation versus Detoxication of the Renal Carcinogen Aristolochic Acid I*: *Studies in cyp1a1(-/-)*, *cyp1a2(-/-)*, *and cyp1a1/1a2(-/-) mice* [25]. The second largest cluster (#1) has 13 members, a silhouette value of 0.879, and is labeled as *occupational kidney disease* by LLR. The most cited author in the cluster is Yang (2011), who wrote *Increased Risks of Upper Tract Urothelial Carcinoma in Male and Female Chinese Herbalists* [26]. In Fig 4D, Cluster #2 is labeled as *screening tool*, Cluster #3 as *aristolochic acid-induced nephropathy*, Cluster #4 as *environment exposure*, and Cluster #6 as *preoperative evaluation*. Cluster #5 (on the right of Fig 4D) has no definite label because this cluster did not have a clear silhouette after clustering. Furthermore, German Canc Res Ctr in Cluster #0 ranked first in terms of citation counts, with 59 citation counts, followed by SUNY at Stony Brook in Cluster #4 with 47 citation counts, Peking University in Cluster #2 with 38 citation counts, King’s College London in Cluster #0 with 32 citation counts, and Charles University, Prague, in Cluster #0 with 30 citation counts.

### Performance of authors

**Fig 5:**
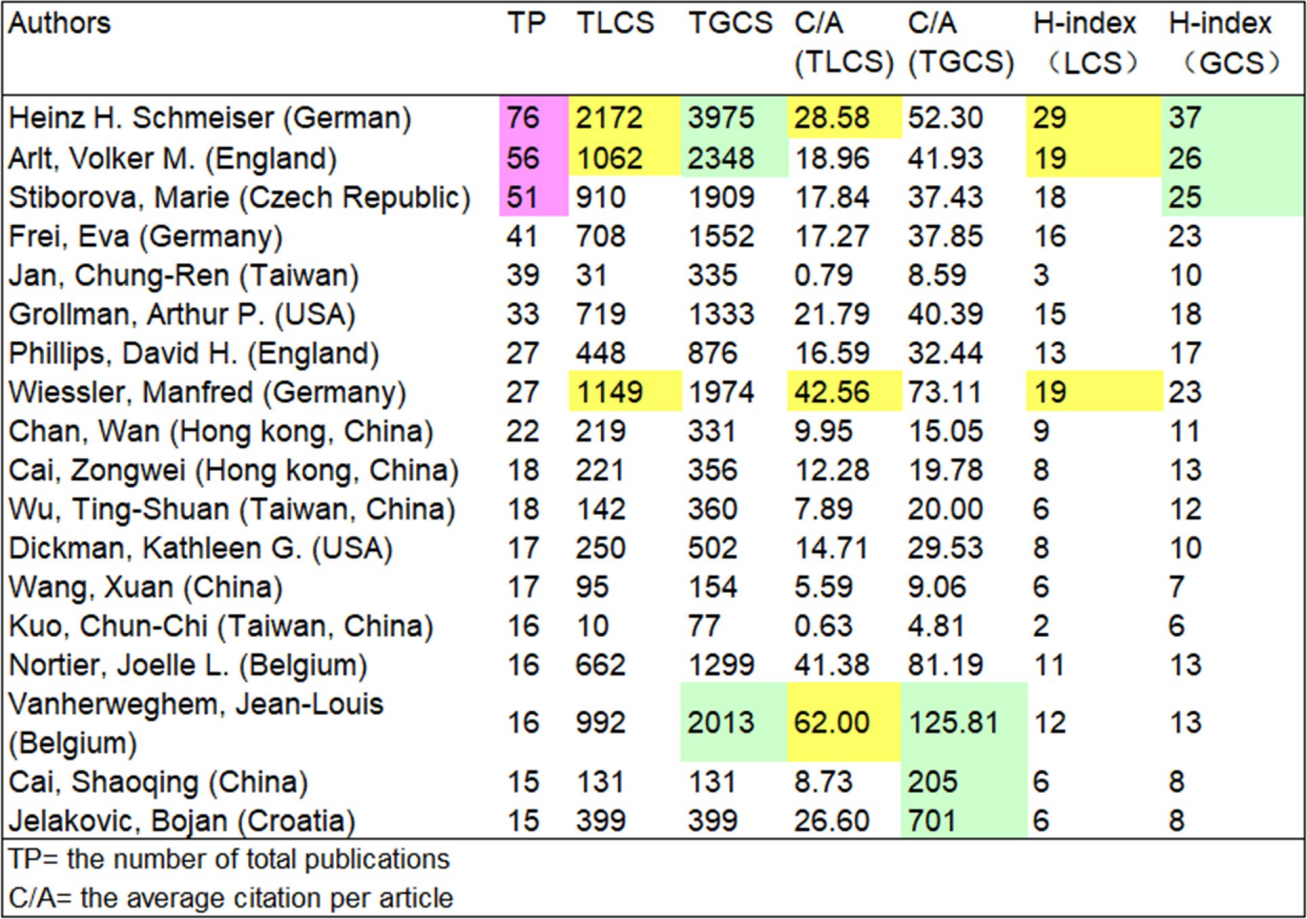
Authors with the highest rate of publication. The cells tinted pink, yellow, and pale green indicate the top three in each column.

The search results showed that 1,217 articles were produced by 4,171 authors. Among them, 178 had five or more publications in the field, with this representing 4.2% of the total number of authors. These authors coauthored with one another in a large proportion of these articles. The top 18 productive authors (where TP, the number of total publications, is greater than 15) are listed in Fig 5. Among them, three were from Germany, and three were from the Taiwan Region. The United States, England, China, Hong Kong, and Belgium each had two productive authors. The German author Schmeiser ranked first, and the performance of other German authors was also impressive, with Frei ranking fourth and Wiessler ranking eighth. Authors who published the most articles on the topic of AA were Schmeiser (Germany), Arlt (England), and Stiborová (Czech Republic), with 76, 56, and 51 publications, respectively. In addition, the GCS-based h-index of their articles also ranked in the top position. Wiessler, who authored 27 articles, was in the top three for TLCS, TLCS-based C/A value, and TLCS-based h-index, whereas Belgium writer Vanherweghem, who authored 16 articles, was in the top three with regard to TGCS, TLCS-based C/A value, and TGCS-based C/A value. Although relatively few articles were produced by Cai (China) and Jelakovic (Croatia), the TGCS of their articles was still extremely high.

### Evolution of research categories

**Fig 6:**
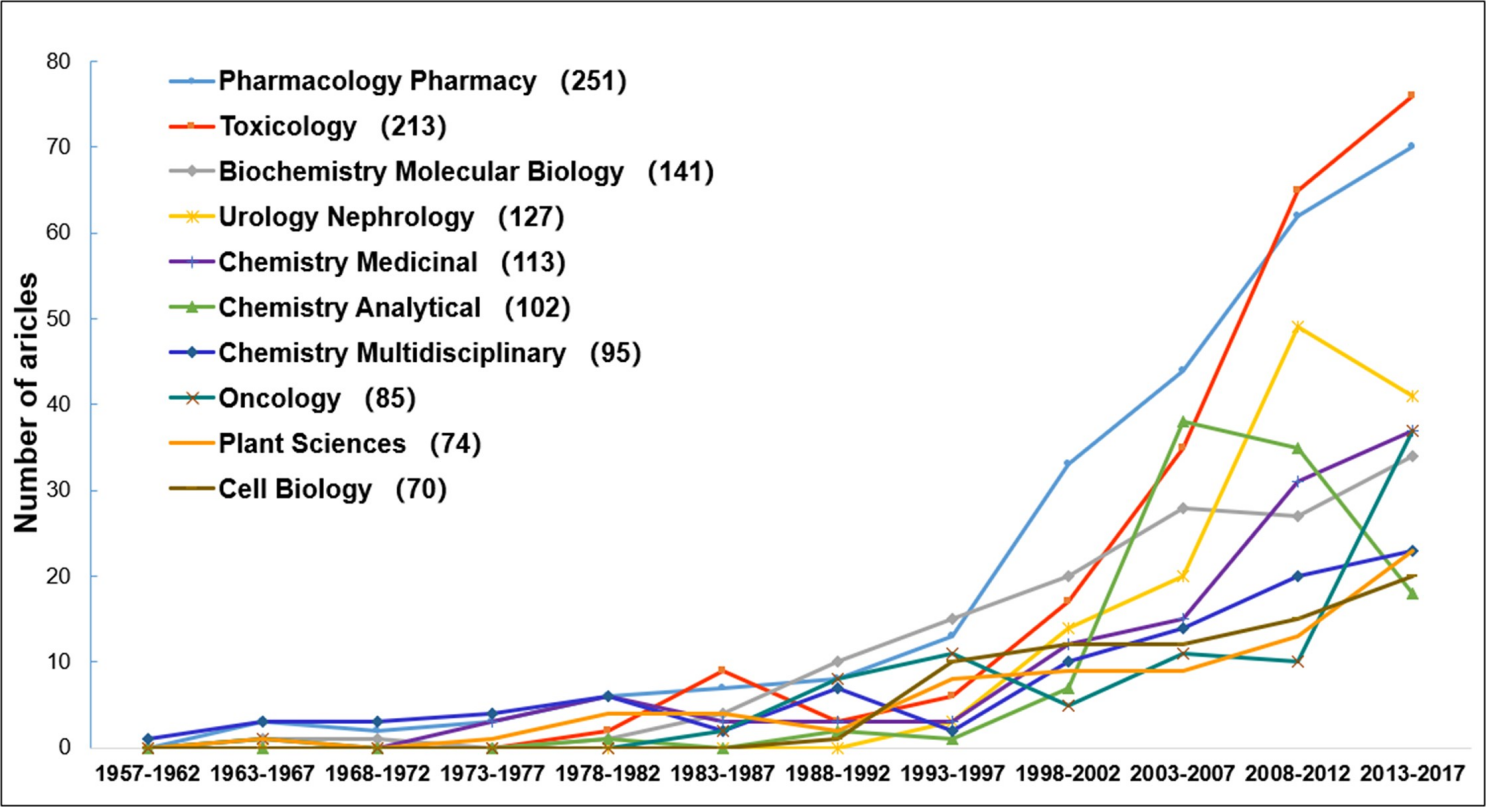
Timeline of articles in the Top 10 most productive categories.

The top 10 research categories are presented in Fig 6. The method used to classify research categories is the classification from the WoS website. Although some articles belong to multiple disciplines simultaneously, this classification method helps us to understand the distribution of articles in various domains throughout the history of AA research. AA-related research has significantly expanded in the past 20 years and is now extensively distributed in different research categories. The trends in their growth over time are shown in Fig 6. As shown in the figure, the topics of *Chemistry Multidisciplinary* and *Pharmacology Pharmacy* were relatively prominent in the initial stage. However, *Pharmacology Pharmacy* expanded rapidly in the late 1990s, together with *Toxicology*, which increased in the late 1980s and had a small upsurge in the mid-1980s. In recent years, *Pharmacology Pharmacy* and *Toxicology* occupied the first two positions of the most productive categories. In contrast, the number of articles in *Chemistry Multidisciplinary* gradually increased. The same phenomenon occurred for *Plant Sciences*, which started early, but slowly increased in number. A steady increase in the number of articles in *Biochemistry Molecular Biology* and *Chemistry Medicinal* was found. The number of articles in *Oncology* increased in the early 1990s and declined slightly at the end of the 20^th^ century but increased rapidly over the past 5 years. The topic *Cell Biology* became more prominent in the late 1990s and has since been growing steadily. Two research categories were found in which the number of articles declined in recent years: *Urology Nephrology*, which slightly declined in the past 5 years; and *Chemistry Analytical*, which rapidly expanded at the beginning of the 21^st^ century, but rapidly declined in the past 10 years.

### Characteristics of scientific outputs

To explore the trends in AA research, we visualized the yearly outputs of relevant articles. The rising trend (time series) of the outputs of AA research in the WoS database between 1957 and 2017 through the basic topic search is shown in Fig 2. Within the years analyzed, the volume of articles follows an overall upward trend. A nonlinear simulation of the time series data revealed that the growth pattern in Fig 2 was close to an exponential function. In addition, the average citations per year per article (ACPP) and the top five publication countries during the period 1957–2017 are also presented in Fig 2. Given the growth trends and ACPP of articles presented in Fig 2, the history of AA research is hypothesized to be divided into three phases, as follows:

- The first phase (1957–1981): the initial stage, during which AA research output increased slowly.
- The second phase (1982–1999): the stage of smooth growth, during which AA research output increased at a constant rate, with a high ACPP in some years.
- The third phase (2000–2017): the period of development during which AA research output increased rapidly.

### Three-phase research focus

#### Keywords

**Fig 7:**
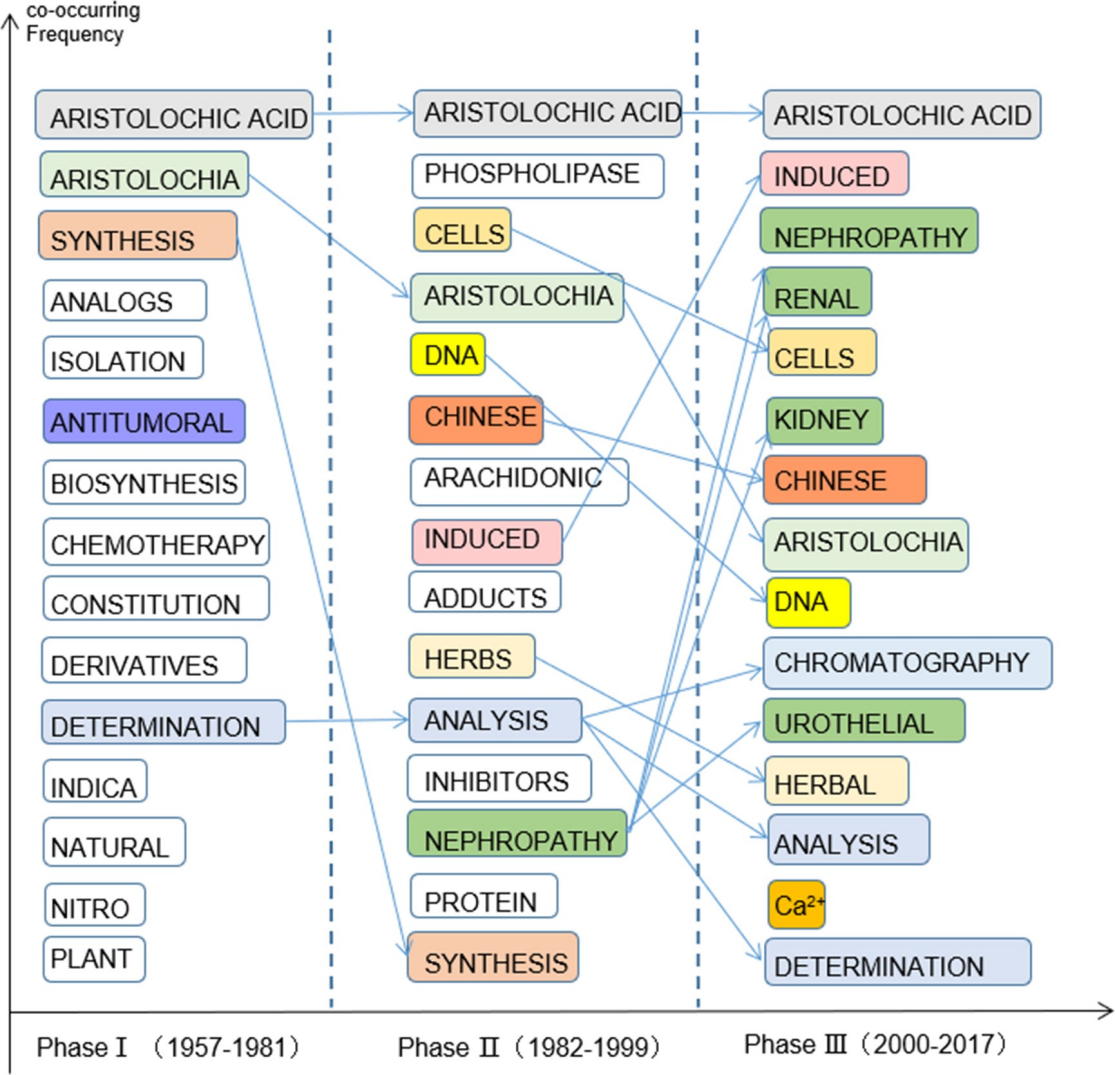
Top 15 most frequent keywords in three phase.

Frequently cited articles and common terms are often utilized to indicate the most well-known scientific hotspots within a specific topic of research. In this study, the 15 most frequent keywords are presented for each phase based on the data produced by HistCite (Fig 7). In combination with the information given in Fig 2, the difference among the three phases appeared to be significant, which could partially support the division into this stages. Thus, the connections and differences between the keywords in all three phases were observed. As for the search words, “aristolochic acid” undoubtedly ranked first in each phase. Some keywords overlapped during the three phases, although the order was sometimes different. For example, the frequencies of the two most frequent keywords (i.e., “aristolochia” and “synthesis”) in Phase I and the two most frequent keywords (i.e., “cells” and “DNA”) in Phase II continuously declined; however, “induced” and “nephropathy” in Phase II increased in number in the subsequent stage. In this statistic, different words under the same category or words with the same meaning may be treated as a group. For example, we treated “nephropathy”, “renal”, and “kidney” as an extension of the word “nephropathy” in Phase II because they are synonyms in Phase III; we treated “chromatography”, “analysis”, and “determination” as an extension of the word “analysis” in Phase II and “determination” in Phase I because they all belong to the analytical chemistry professional vocabulary. Keywords that belong to the chemical category analysis were always in a relatively low position (Fig 7).

Keywords all belonged to the chemistry category in Phase Ⅰ, such as “synthesis”, “analogs”, “isolation”, and “biosynthesis”. In addition, “antitumoral” is a special keyword in Phase Ⅰ because it is the only word describing the pharmacological activity of AA in the early stage in the top 15 most frequent keywords, which shows that AA was considered a potential antitumor agent from the initial stages of research. In comparison with Phase I, keywords about the mechanism of the toxicology of AA emerge in Phase II (i.e., “DNA”, “induced”, and “adducts”). Furthermore, words related to the immune activity of AA (e.g., “phospholipase”, “inhibitors”, and “protein”) emerge in Phase II, although they are not all in the top 15 most frequent words in Phase III. “Ca^2+^” only occurs in the top 15 in Phase III. An article in 2017 indicated that as an inhibitor of PLA2, AA could significantly decrease the arachidonic acid-induced changes in intracellular Ca^2+^ concentration in round spermatids [27].

#### Highlighted articles and journals

To further explore the differences among the three phases in detail and illustrate some facets of the evolutionary trend of the relevant research on AA, a historical overview of each phase was conducted (Tables 1 and 2).

**Table 1:** Top 10 studies most frequently cited in each phase (1957–2017).

**Table 1a. Phase I (1957–1981).**								
**Rank**	**Title**	**LCS**	**GCS**	**First author**	**Year**	**Journal**	**IF**	**Ref.**
1	Isolation and Structural Elucidation of Novel Derivatives of Aristolochic Acid from Aristolochia Indica	30	56	Kupchan, SM	1968	Journal of Organic Chemistry	4.849	[33]
2	Aristolochic Acids from Aristolochia-Manshuriensis	24	31	Rucker, G	1975	Planta Medica	2.342	[35]
3	Tumor Inhibitors X. Photochemical Synthesis of Phenanthrenes. Synthesis of Aristolochic Acid and Related Compounds	21	115	Kupchan, SM	1965	Journal of Organic Chemistry	4.849	[30]
4	Aristolochic Acid-I in Swallowtail Butterfly Pachlioptera Aristolochiae (Fabr) (Papilionidae)	14	33	Voneuw, J	1968	Israel Journal of Chemistry	2.455	[32]
5	Aristolochic Acids Stored by Zerynthia-Polyxena (Lepidoptera)	14	29	Rothschild, M	1972	Insect Biochemistry	3.756	[93]
6	Constitution of Aristolochic Acids 3 and 3A - (Plant Constituents Containing a Nitro Group V.	11	17	Pailer, M	1966	Monatshefte Fur Chemie Und Verwandte Teile Anderer Wissenschaften	_	[94]
7	Isolation of aristolochic acid from two Madagascar Aristolochiaceae. Determination of its toxicity on vegetal cells. Comparison with animal cells (Author’s translation)	11	13	Moretti, C	1979	Planta Medica	2.342	[37]
8	Aristolochic Acid Intoxication—A New Type of Impairment of Urinary Concentration Ability	10	22	Peters, G	1963	Archives Internationales De Pharmacodynamie Et De Therapie	0.72	[29]
9	Further Studies on Aristolochic Acid. 1. Communication	10	9	Mose, JR	1974	Arzneimittel-Forschung/Drug Research	_	[95]
10	The Chemistry of the Aristolochia Species. 3. Aristolochic Acids and Related Substances from Aristolochia-Reticulata and A-Indica	9	14	Coutts, RT	1957	Journal of the Chemical Society	0.327	[28]
**Table 1b. Phase II (1982–1999).**								
**Rank**	**Title**	**LCS**	**GCS**	**First author**	**Year**	**Journal**	**IF**	**Ref.**
1	Rapidly Progressive Interstitial Renal Fibrosis in Young-Women—Association with Slimming Regimen Including Chinese Herbs	327	649	Vanherweghem, JL	1993	Lancet	43.831	[8]
2	Detection of DNA Adducts Formed by Aristolochic Acid in Renal Tissue from Patients with Chinese Herbs Nephropathy	143	221	Schmeiser, HH	1996	Cancer Research	9.122	[42]
3	The Carcinogenic Action of Aristolochic Acid in Rats	109	151	Mengs, U	1982	Archives of Toxicology	5.901	[38]
4	Chinese Herbs Nephropathy—A Clue to Balkan Endemic Nephropathy	102	182	Cosyns, JP	1994	Kidney International	8.395	[44]
5	Urothelial Lesions in Chinese-Herb Nephropathy	101	186	Cosyns, JP	1999	American Journal of Kidney Diseases	7.623	[45]
6	P-32-Post-Labelling Analysis of DNA Adducts Formed by Aristolochic Acid in Tissues from Patients with Chinese Herbs Nephropathy	98	81	Bieler, CA	1997	Carcinogenesis	5.105	[43]
7	Studies on The Metabolism of Aristolochic Acid-I and Acid-II	70	86	Krumbiegel, G	1987	Xenobiotica	1.932	[96]
8	Aristolochic Acid Binds Covalently to The Exocyclic Amino Group of Purine Nucleotides In DNA	68	96	Pfau, W	1990	Carcinogenesis	5.105	[41]
9	Quantitative Analysis of Aristolochic Acids, Toxic Compounds, Contained in Some Medicinal Plants	61	83	Hashimoto, K	1999	Journal of Ethnopharmacology	2.981	[97]
10	DNA Adduct Formation of Aristolochic Acid-I And Acid-II Invitro and i*n vivo*	59	77	Schmeiser, HH	1988	Carcinogenesis	5.105	[40]
**Table 1c. Phase III (2000–2017).**								
**Rank**	**Title**	**LCS**	**GCS**	**First author**	**Year**	**Journal**	**IF**	**Ref.**
1	Urothelial Carcinoma Associated with the Use of a Chinese Herb (Aristolochia fangchi).	234	603	Nortier JL	2000	New England Journal of Medicine	72.406	[46]
2	Aristolochic Acid and the Etiology of Endemic (Balkan) Nephropathy	179	315	Grollman AP	2007	Proceedings of the National Academy of Sciences of the United States of America	9.661	[11]
3	Aristolochic Acids Induce Chronic Renal Failure with Interstitial Fibrosis in Salt-Depleted Rats	102	143	Debelle FD	2002	Journal of the American Society of Nephrology	8.966	[48]
4	Aristolochic Acid-Associated Urothelial Cancer in Taiwan	76	158	Chen CH	2012	Proceedings of the National Academy of Sciences of the United States of America	9.661	[51]
5	Chronic Aristolochic Acid Toxicity in Rabbits: A Model of Chinese Herbs Nephropathy?	66	103	Cosyns JP	2001	Kidney International	8.395	[98]
6	Human Enzymes Involved in the Metabolic Activation of Carcinogenic Aristolochic Acids: Evidence for Reductive Activation by Cytochromes P450 1A1 and 1A2	63	115	Stiborová M	2001	Chemical Research in Toxicology	3.278	[55]
7	Aristolochic Acid Mutagenesis: Molecular Clues to the Aetiology of Balkan Endemic Nephropathy-Associated Urothelial Cancer	62	96	Arlt VM	2007	Carcinogenesis	5.105	[49]
8	Rapidly Progressive Fibrosing Interstitial Nephritis Associated with Chinese Herbal Drugs	60	109	Yang CS	2000	American Journal of Kidney Diseases	7.623	[47]
9	Structure Activity Relationships of Aristolochic Acid Analogues: Toxicity in Cultured Renal Epithelial Cells	60	77	Balachandran P	2005	Kidney International	8.395	[99]
10	Carcinogenic Aristolochic Acids Upon Activation by DT-Diaphorase form Adducts Found in DNA of Patients with Chinese Herbs Nephropathy	59	89	Stiborová M	2002	Carcinogenesis	5.105	[56]

**Table 2:** Top 8 journals with the highest IF (IF>10) in phase III (2000–2017).

Rank	Journal	IF	Title	First author	Year	LCS	GCS	Ref.
1	New England Journal of Medicine	72.41	Urothelial Carcinoma Associated with the Use of a Chinese Herb (Aristolochia Fangchi)	Nortier, JL	2000	234	603	[46]
2	Lancet	47.83	Urothelial Malignant Disease and Chinese Herbal Nephropathy	Lord, GM	2001	47	125	[52]
			Chronic Kidney Disease: Global Dimension and Perspectives	Jha, Vivekanand	2013	4	719	[53]
			Global Kidney Health 2017 and Beyond: A Roadmap for Closing Gaps in Care, Research, and Policy	Levin, Adeera	2017	0	5	[54]
3	Nature Biotechnology	41.67	Rat Toxicogenomic Study Reveals Analytical Consistency Across Microarray Platforms	Guo, Lei	2006	6	280	[100]
4	Science Translational Medicine	16.8	Genome-Wide Mutational Signatures of Aristolochic Acid and Its Application as a Screening Tool	Poon, Song Ling	2013	0	60	[16]
			Mutational Signature of Aristolochic Acid Exposure as Revealed by Whole-Exome Sequencing	Hoang, Margaret L	2013	0	53	[61]
			Aristolochic Acids and their Derivatives are Widely Implicated in Liver Cancers in Taiwan and Throughout Asia	Alvin W. T. Ng	2017	0	2	[1]
5	European Urology	16.26	Select Screening in a Specific High-Risk Population of Patients Suggests a Stage Migration Toward Detection of Non-Muscle-Invasive Bladder Cancer	Zlotta, Alexandre R	2011	2	15	[101]
6	Journal of the National Cancer Institute	12.59	Population-Based Case-Control Study of Chinese Herbal Products Containing Aristolochic Acid and Urinary Tract Cancer Risk	Lai, Ming-Nan	2010	46	111	[50]
7	Nature Communications	12.12	Variation in Genomic Landscape of Clear Cell Renal Cell Carcinoma Across Europe	Scelo, Ghislaine	2014	0	41	[62]
			Mutational Landscape of Intrahepatic Cholangiocarcinoma	Zou, Shanshan	2014	0	45	[66]
8	Nucleic Acids Research	10.16	DNA Adducts of Aristolochic Acid II: Total Synthesis and Site-Specific Mutagenesis Studies in Mammalian Cells	Attaluri, Sivaprasad	2010	23	35	[57]
			Lack of Recognition by Global-Genome Nucleotide Excision Repair Accounts for the High Mutagenicity and Persistence of Aristolactam-DNA Adducts	Sidorenko, Victoria S.	2012	23	49	[58]
			Structure and Stability of DNA Containing an Aristolactam II-dA Lesion: Implications for the NER Recognition of Bulky Adducts	Lukin, Mark	2012	9	20	[59]
			Adenine Versus Guanine DNA Adducts of Aristolochic Acids: Role of the Carcinogen-Purine Linkage in the Differential Global Genomic Repair Propensity	Kathuria, Preetleen	2015	0	4	[60]

Articles in Phase I (1957–1981) mostly focused on chemistry, and AAs were actually regarded as tumor inhibitors in the early stage. The main information was summarized based on the bibliometric results from HistCite and the information from in Table 1A and Fig 2. The outputs per year and ACPP, as shown in Fig 2, were low in Phase I. The first article of this phase, which was the first in the history of AA research, was published in 1957 [28] and belonged to the phytochemistry category. In 1963, researchers identified the toxicity of AA for the first time [29]; however, prior to that, the research has not attracted much attention, and the LCS and GCS of the research were relatively low. The ACPP of the 1965 was relatively high, which may be because the first scientific research in chemical synthesis field about AA was published in 1965 [30]. In contrast, the first biosynthesis field paper about AA was published in 1967 [31], but had attracted relatively low attention (that is, the LCS was 6 and the GCS was 12, and not in the top 10). The ACPP in 1968 was high, which may be because two well-known articles were published in that year, both of which belonged in the phytochemistry field [32, 33]. The semi-synthesis of AA was first reported in 1975; however, it did not receive considerable attention [34]. Another article published in 1975 belonged to the field of phytochemistry [35]. In 1978, the first article about AA in the analytical chemistry field was published, in which a polarographic determination of AA was reported [36]. In 1979, scientists reported the cytotoxicity of AA to animal tumor cells and vegetal cells in vitro [37]. This finding indicated that AA was regarded as potential tumor inhibitor in the early stage of research, which was consistent with the results of the keyword analysis.

Since 1982 (the beginning of Phase II), the LCS and GCS of articles about AA research increased sharply (Fig 2), and the toxicity of AA was acknowledged extensively by scientists (Table 1B). The first famous article was published in 1982 in the *Archives of Toxicology*, which reported the carcinogenic action of AA in rats [38]. Notably, in 1985, scientists reported that AA induced 6-thioguanine-resistant mutants in the extrahepatic tissue of rats after oral application; and dose-dependent mutagenic activity was observed [39]. However, this scientific research did not attract attention (that is, LCS of 3 and GCS of 13). In 1988, Schmeiser *et al*. analyzed the DNA adduct formation using a ^32^P-post-labeling assay and found that the DNA adduct formation by AA I and AA II did not directly correlate with the initiation of the carcinogenic process and the subsequent tumor formation in target tissues [40]. Since then, the enthusiasm for research into AA-DNA adducts has intensified [41–43]. In 1993, an article published by Vanherweghem *et al*. received the highest citation rate [8]. In this study, the authors reviewed the inadvertent treatment with AA-containing herbs that caused kidney failure in patients at a Belgian weight loss clinic in the early 1990s. In 1994, the term “Chinese herb nephropathy” (CHN) was mentioned for the first time [44]. Cosyns *et al*. indicated that on morphological and clinical grounds, CHN was similar to Balkan endemic nephropathy (BEN) and a common etiologic agent, AA, was suspected. Patients with CHN were suggested to undergo a regular follow-up for urothelial malignancy based on the known carcinogenic potential of AA and the multiple foci of cellular atypia of the urothelium, which was found by the authors. Then, in 1999, Cosyns *et al*. found that AA-related carcinogenesis was associated with the overexpression of p53 [45].

The third stage (2000–2017) has seen explosive growth in the number of articles published. During this period, an increasing number of experts and scholars from different countries and regions has devoted themselves to AA-related research. The role of Chinese scientists is of note (Fig 2). The top 10 most cited articles in Phase III are listed in Table 1C. It is common that the citation rate is lower when articles are newly published, because citations accumulate over time and may lag behind the true value of the article. Therefore, when we analyzed the articles in Phase III, we also compiled the articles published in journals with a high impact factor (IF>10; the impact factor of a given journal is determined as reported in the 2017 *Journal Citation Reports*) in Table 2, separately from Table 1C. After combining the information in Tables 1C and 2, we summarized the information based from the following perspectives. First, articles published in journals with a high impact factor were cited more frequently. The article published in the *New England Journal of Medicine* by Nortier *et al*. in 2000 with an impact factor of 72.406 is a good example; this study proposed that the cumulative dose of aristolochia was a significant risk factor for urothelial carcinoma, with total doses of more than 200 g associated with a high risk of urothelial carcinoma [46]. Second, scholars have become increasingly concerned about AA-induced diseases, particularly in the urinary system [11, 47–51]. This information is consistent with Fig 7, in which the usage of keywords “nephropathy”, “renal”, “kidney”, and “urothelial” in Phase III increase. Between 2001 and 2017, *Lancet* has published three AA-related articles [52–54]. At present, experts have observed minimal success for the identification of the root causes of chronic kidney disease, which includes recurrent acute kidney injury; heat stress; dehydration; infections; exposure to agrochemicals, over-the-counter medications; heavy metal contamination; poor quality drinking water; and other combinations thereof [54]. Third, the mechanism of AA in triggering diseases is another research hotspot. In 2001, Stiborová *et al*. reported the activation of AA by human enzymes, and their results clearly demonstrated the role of P450 1A1, 1A2, and NADPH: P450 reductase in catalyzing the reductive activation of AA [55]. In 2002, this research group reported a reductive activation of carcinogenic AAs by DT-diaphorase [56]. *Nucleic Acids Research* has published four relevant articles in recent years [57–60]. In 2015, Kathuria *et al*. published a study that utilized computational modeling to provide a plausible structural explanation for the experimentally observed differential global genome repair propensity of the ALII-N-2-dG and ALII-N-6-dA DNA adducts of AA II [60]. Finally, scientists are also interested in the exploration of the scientific significance of mutational signature induced by AA exposure; three related articles have been recently published in *Science Translational Medicine* magazine [1, 16, 61] (Table 2). In 2017, Ng *et al*. found that the mutational signature of AA exposure was geographically widespread: Asia, especially in the Taiwan Region, appeared to be considerably more extensively affected, which was consistent with other evidence on patterns of AA exposure [1]. Several groups independently and reproducibly detected a consistent AA mutational signature in hepatocellular carcinomas (HCCs) [16], upper tract urothelial cancers (UTUCs) [16], intrahepatic bile duct carcinomas (IBDCs) [62], renal cell carcinomas (RCCs) [63], bladder carcinomas (BCs)[64], and bile duct cancers (BDCs) [65, 66]. Nevertheless, Ng *et al*. stated that they could not exclude the possibility that chemicals unrelated to AAs, aristolactams, and their derivatives might also induce a mutational signature similar to that induced by AAs; however, at present, no such chemical is known.

**Fig 8:**
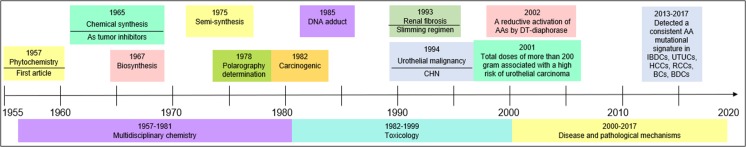
Roadmap of the historical development of AA-related research.

Based on the above analysis, a roadmap of the historical development of the research about AA was established to present the results in an intuitive manner (Fig 8).

### Analysis of review articles

**Fig 9:**
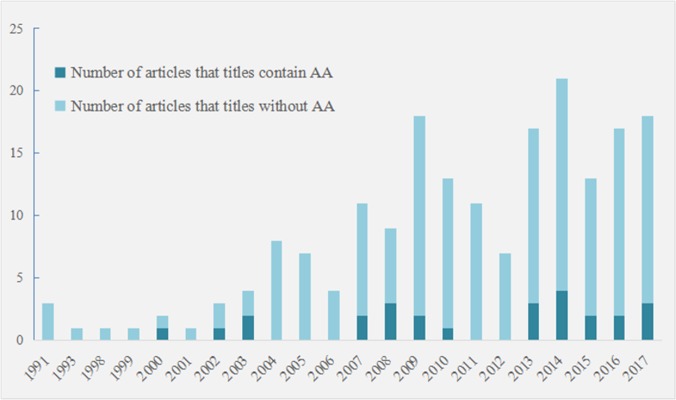
Number of AA-related review articles.

To the end of December 2017, 189 AA-related review articles were retrieved from searches of the WoS database; 26 were directly related to AA, as characterized by the inclusion of AA in the title. These review articles written by domain experts are outlined in Fig 9 to allow comparison with the outputs of the prior analyses.

The first review article related to the theme of AA was published in 1991 [67]; however, the first review article directly related to AA (with a title containing AA) was published in *Chemické Listy* in 2000, and explained the molecular mechanism of a unique type of renal fibrosis designated as CHN [68]. In 2002, Arlt *et al*. conducted a deep review on the genotoxic and carcinogenic mechanism of AA in rodents, as well as the nephrotoxic and carcinogenic mechanisms of AA in humans; they suggested that all products containing AA should be banned from the global market [69]. As the number of relevant articles focused on the toxicological mechanism increased in 2003, the first review of natural products emerged. Kumar *et al*. reviewed and classified naturally occurring aristolactams, AAs, and dioxoaporphines based on their oxygenation pattern [70]. Furthermore, two articles related to the topic of natural products were released in 2011 [71] and 2014 [72]; these indicated the urgent need for scholars to make a thorough overview on the naturally occurring AA discovered in recent years to keep up with the progress of scientific research in this area.

Between 2004 and 2006, 19 AA-related review articles were published, which was greater than the total number of articles published between 1991 and 2003. The titles of review articles published in this stage do not contain AA; nevertheless, the content is, to some extent, related to AA. Some review articles published in 2004 focused on the toxicity of herbal medicines owing to their wide use and ease of availability, and gradually revealed their safety [73, 74]; moreover, the quality control of herbal medicines became a higher priority [75, 76]. In 2005, CHN and AA nephropathy were extensively discussed by Colson and De Broe, who explored kidney injury from complementary or alternative medicines [77]. In 2006, Stefanovic *et al*. performed an etiology analysis of BEN and associated urothelial cancer. Notably, the AA hypothesis is one of the three theories that attempt to explain the environmental cause of the familial chronic tubulointerstitial disease BEN; thus, the investigation of gene–gene and gene–environment interactions could be the content of further studies to determine the precise risk of BEN [78].

Over the past decade, AA-related diseases have received considerable attention. In total, the number of articles has considerably increased since 2007; in only two years (2008 and 2012) were fewer than 10 articles published. From 2007 to 2012, five review articles were related to kidney disease, seven to urothelial carcinoma, and 14 to carcinoma or cancer solely. In contrast, from 2013 to 2017, 11 review articles related to kidney disease were published, 14 related to urothelial carcinoma, and 34 to the cancer solely. The number of review articles that are relevant to various types of cancer has increased in this area of research, which partly supports the previous conclusion drawn from the analysis of keywords and articles that scholars are becoming increasingly concerned about AA-related diseases.

Since the discovery of the toxicity of AA, the discussion on its mechanisms has never stopped. Given the increased body of research, several review articles have emerged. Some are mainly relevant to the formation of DNA adducts, is are thought to be the primary mechanism of AA-driving carcinogenesis [79–81]; others have focused on the monitoring techniques of adducts [82–84]. In 2017, two relevant review articles were published. As the title implied, Hollstein *et al*. believed that “*base changes in tumor DNA have the power to reveal the causes and evolution of cancer”* and indicated that the tasks now confronting the field of molecular epidemiology are to assign mutagenic processes to orphan and newly discovered tumor mutation patterns and determine whether avoidable cancer risk factors influence the signatures produced by endogenous enzymatic mechanisms [85]. Stiborová *et al*. showed that differences in AA metabolism might be one of the reasons for an individual’s susceptibility in the multistep process of AA carcinogenesis and that studying the associations between the activities and/or polymorphisms of the enzyme metabolizing AA is an important determinant in the identification of individuals with have a high risk of the development of AA-mediated upper urothelial cancer. Therefore, “*DNA adducts formed by AA are unique biomarkers of exposure and explain the initiation phase of upper urothelial cancer*” [86].

In addition to knowledge of AA toxicology, researchers have also found that AA is immunologically active. Since 1993, several review articles on the inhibitory properties of AA against snake venoms have emerged [87–91]. However, four review articles related to the theme of inflammation were published in 2007, three in 2013, but only one in 2016. Recent efforts in this research in this area are contracting, so experts should concentrate on this specific theme. Moreover, the study of quality control should be strengthened as scholars deepen their understanding of toxicology. Nevertheless, the number of articles published on the theme of analytical chemistry has declined significantly over the past decade (Fig 6). The quality control of toxic substances is imminent, and detection technology is particularly important. In 2011, an article reviewed the advances on HPLC/MS in medicinal plant analysis (2006–2011), in which AA-related research has been summarized [92]. Subsequently, no other review articles have focused on the detection techniques for AA and DNA adducts.

## Conclusions

This current study is the first comprehensive review of the various aspects of AA-related studies using bibliometric methods. In total, 1,217 articles and 189 review articles related to AA research between 1957 and 2017 have been analyzed. Five clear suggestions are presented and can be summarized as follows: (1) The anti-tumor efficacy of aristolochic acids is a valuable topic for study. Indeed, researchers discovered the tumor-suppressive activity of AA in the early stages of research and eventually slowed because of the identified toxicity of AA. However, structural modification, Chinese medicine processing methods, or some other biological methods should be considered. (2) The immune activity of AAs should also be further explored. Given the results of the keyword analysis in Phase II and for review articles, the immune activity of AAs and their derivatives acting as snake venom inhibitors is an important research field in Phase II. According to Chinese medicine theory, herbs containing AA often have the effect of heat-clearing and detoxifying. The immune activity of AA may provide an explanation for this theory. (3) Scholars should make a thorough overview on the discovery of naturally occurring AAs owing to their toxicity and wide distribution in various plants. (4) More efforts should be directed toward exploring the correlation between the mutational signature of AA and various cancers, because the existence of such connections has not yet reached a definite conclusion. In contrast, there is insufficient epidemiological and animal experimental evidence to support a direct correlation between the mutational signature of AA and cancer; however, it is not yet known whether there are other factors contributing to the mutational signature of AA. (5) Further efforts should be devoted to the research and review work in the theme of analytical chemistry. Additional and better rapid detection technologies are needed. The detection techniques for AA in plant samples and AA adducts for biological samples have yet to be improved.

The following are some of the other most notable findings:

- The output of Mainland China was the highest, and articles from the United States have the highest quality. Switzerland and Austria have maintained the most active collaborations.
- German Canc Res Ctr is the most productive institution, followed by SUNY at Stony Brook in the United States. After a bibliographic coupling analysis of institutions, seven clusters emerged; German Canc Res Ctr is part of in the largest cluster (Cluster #0), labeled as *carcinogenic aristolochic acid*.
- The most productive author is Schmeiser from Germany, whose articles also obtained the highest citation score and h-index (based on TLCS and TGCS).

- *Pharmacology Pharmacy* and *Toxicology* are the two most extensively distributed research categories of articles in this specific field in recent years; however, the number of articles in *Chemistry Analytical* has rapidly decreased in the last 10 years.
- The history of AA research is hypothesized to have three phases. Overall, Phase I (1957–1981) is the starting stage; Phase II (1982–1999) is the stage during which the number of articles increased slightly; and Phase III (2000–2017) is the stage of explosive growth in the number of articles. In Phase I, the articles mostly focused on chemistry, whereas in Phase II, researchers began to pay attention to pharmacology and toxicology and the term CHN was first mentioned. In Phase III, scholars have become increasingly concerned about AA-induced diseases and focused on research into diseases at the molecular level.
- The analysis of the review articles has indicated that most of the articles are about the pathology and mechanism of AA-induced urinary system diseases; the number of review articles on AA immunoreactivity in recent years is smaller, which was similar to the review articles in analytical chemistry category.

The findings obtained from the bibliometric analysis and traditional review are expected to benefit researchers in shaping their future research direction and achieving further innovative ideas. However, the researchers should consider several limitations of this study. (1) The periodization method may not be scientific and may be overturned because it is a hypothesis. (2) The citation count of the articles may only reflect the impact of the articles in a quantitative manner, but not in a qualitative manner. (3) Given the large quantity of data, this study did not fully investigate whether there are articles that cite AA-related research, but do not contribute to the study of AA.

## Abbreviations

AAs: aristolochic acids
AA: aristolochic acid
WoS: Web of Science
TLCS: total local citation score
LCS: local citation score
TGCS: total global citation score
GCS: global citation score
LLR: log-likelihood ratio
TP: the number of total publications
C/A: average citation per article
ACPP: average citations per article per year
CHN: Chinese herb nephropathy
BEN: Balkan endemic nephropathy
IF: impact factor
HCCs: hepatocellular carcinomas
UTUCs: upper tract urothelial cancers
IBDCs: intrahepatic bile duct carcinomas
RCCs: renal cell carcinomas
BCs: bladder carcinomas
BDCs: bile duct cancers
